# The Facial Feedback Effect: Consistent Across Ages, Divergent Across Emotions

**DOI:** 10.1093/geroni/igaf122.2912

**Published:** 2025-12-31

**Authors:** Nikolina Kravljaca, Jennifer Stanley

**Affiliations:** The University of Akron, Akron, Ohio, United States; The University of Akron, Akron, Ohio, United States

## Abstract

Emotions are more than just a psychological experience; they are expressed through the body in ways that influence how emotions are felt. This bidirectional relationship between mind and body reflects embodied cognition, which suggests that physical expressions are not merely passive reflections of emotion but active contributors (Costello & Bloesch, 2017). One example is the facial feedback effect, where forming a facial expression, such as a smile, can elicit changes in emotional state (James, 1890). Few studies have examined whether the facial feedback effect operates similarly in older adulthood. This study investigated potential age differences in facial feedback effects by comparing young (n = 69) and older adults’ (n = 65) emotional ratings while holding posed expressions. Participants posed facial expressions (happy, angry, or neutral) while completing an emotion-rating questionnaire assessing feelings of happiness and anger. Contrary to our hypotheses, both young and older adults reported increased anger when scowling, but no significant changes in happiness while smiling. Young adults’ (but not older adults’) baseline anger scores positively correlated with their anger ratings after posing an angry facial expression. Coupled with the relatively low ratings of anger overall, future work should investigate whether individual differences in trait-level emotions moderate facial feedback effects. These findings suggest facial feedback effects are not exclusive to young adults; however, additional emotional context may be necessary to reliably influence the emotional experience. These results contribute to a growing understanding of how physical expression and emotional experience interact across the lifespan.

